# Advancing Human Vaccine Development Using Humanized Mouse Models

**DOI:** 10.3390/vaccines12091012

**Published:** 2024-09-04

**Authors:** Runpeng Han, Lishan Su, Liang Cheng

**Affiliations:** 1 Department of Infectious Diseases, Zhongnan Hospital of Wuhan University, Medical Research Institute, Frontier Science Center for Immunology and Metabolism, Wuhan University, Wuhan 430071, China; 2 Center for AIDS Research, Wuhan University, Wuhan 430071, China; 3 Laboratory of Viral Pathogenesis and Immunotherapy, Institute of Human Virology, Department of Pharmacology, University of Maryland School of Medicine, Baltimore, MD 02121, USA

**Keywords:** human vaccine, infectious diseases, humanized mice, HIV-1

## Abstract

The development of effective vaccines against infectious diseases remains a critical challenge in global health. Animal models play a crucial role in vaccine development by providing valuable insights into the efficacy, safety, and mechanisms of immune response induction, which guide the design and formulation of vaccines. However, traditional animal models often inadequately recapitulate human immune responses. Humanized mice (hu-mice) models with a functional human immune system have emerged as invaluable tools in bridging the translational gap between preclinical research and clinical trials for human vaccine development. This review summarizes commonly used hu-mice models and advances in optimizing them to improve human immune responses. We review the application of humanized mice for human vaccine development with a focus on HIV-1 vaccines. We also discuss the remaining challenges and improvements needed for the currently available hu-mice models to better facilitate the development and testing of human vaccines for infectious diseases.

## 1. Introduction

Infectious diseases remain a significant global health burden, causing substantial morbidities and mortalities across populations worldwide. Vaccination campaigns are one of the most effective public health efforts in combating infectious diseases. In certain cases, traditional vaccines, such as live attenuated or inactivated vaccines and protein-based subunit vaccines, provide long-lasting immunity: for instance, yellow fever [1], smallpox [2], and hepatitis B virus vaccines [3]. However, prophylactic or therapeutic vaccines for many other pathogens, such as HIV-1 and mycobacterium tuberculosis, are not yet available.

Animal models are essential tools in the development of vaccines, offering insights into vaccine efficacy, safety, and mechanisms of immune response induction, which inform vaccine design and formulation. Various animal models, ranging from rodents to non-human primates (NHPs) are utilized for the development and testing of vaccines. However, preclinical data generated in animal models are not always reliable when translated into clinical trials. In NHPs whose genomes are approximately 98% identical to the human genome, small differences can significantly affect the pathogenesis and immune responses to viruses (reviewed in ref. [4]). For instance, chimpanzees share no human leukocyte antigen (HLA) class I alleles with humans, and distinct HLA class II differences were observed as well [4,5]. These differences may impede the utilization of NHPs in vaccine development and testing, especially T cell-based vaccines, as the activation of T cells is controlled by T cell receptor (TCR) engagement with antigen-presenting HLA molecules. In addition, many pathogens such as HIV-1 yield unique human tropism, resulting in the inability to investigate disease hallmarks and immune responses in animal models.

Mice reconstituted with human immune systems (hu-mice) enable the study of immune responses to human-tropic pathogens and vaccines in vivo, facilitating the evaluation of B cell- and T cell-based vaccine candidates against infectious agents such as HIV-1 [6,7,8,9,10,11,12,13,14,15,16] and plasmodium parasites [17] in a physiologically relevant context. Here, we discuss the currently in-use humanized models and advances that have been made in vaccine development utilizing these models, with a focus on vaccines against HIV-1.

## 2. Brief History of the Hu-Mice Models

The identification of immunodeficient mice enabled the study of human cells in mouse models (Table 1). The SCID-hu PBL mice generated by engrafting human peripheral blood leukocytes (PBLs) into C.B.-17-SCID mice, which lack both T and B lymphocytes, marked the first breakthrough in the field [18]. Following immunization with tetanus toxoid, the reconstituted SCID-hu PBL mice mounted an antigen-specific human antibody response [18]. The SCID-hu PBL mice also enable researchers to study human T cell responses to certain antigens to inform T cell-based vaccine designs. For instance, Okada et al. tested SARS-N and SARS-M DNA vaccines and observed strong cytotoxic T lymphocyte (CTL) expansion and effector cytokine production in SCID-hu PBL mice [19]. Yoshida et al. injected inactivated HIV-1-pulsed autologous dendritic cells (DCs) into the spleens of SCID-hu PBL mice and showed that this vaccination strategy provides protection against R5 HIV-1 infection [6]. Non-obese diabetic (NOD)/SCID mice, which lack T and B cells, and which have compromised NK cell and macrophage function [20], were used for their superior accommodation of human PBMCs [21] and hematopoietic stem cell (HSC) [22] accommodation. However, the lack of human lymphoid organs and the xeno-reactive T-cell-mediated graft-versus-host diseases limited the application of early NOD/SCID-hu PBL or NOD/SCID-hu HSC models [23].

With the evidence that the depletion of mouse NK cells increases the engraftment efficiency of human HSC [31], researchers turned to IL-2 receptor gamma chain-deficient mice, where mouse NK cell development is totally ablated due to deficiency in IL-7 and IL-15 signaling. Multilineage hematopoiesis, including monocytes, DCs, erythrocytes, platelets, T cells, and B cells, was observed after engrafting human HSCs into NOG (NOD.Cg-*Prkdc^scid^Il2rg^tm1Sug^*/ShiJic) mice, where the intracellular signaling domain of the common γ chain is deleted [26], or NSG (NOD.Cg-*Prkdc^scid^Il2rg^tm1Wjl^*/SzJ) mice, where common γ chain expression is completely annulled [27,32]. The discovery of recombination-activating genes (RAGs) 1 and 2, and their roles in T and B lymphocyte receptor rearrangement, led to the development of two other immunodeficient mice models, *Rag1*^−/−^ and *Rag2*^−/−^ mice, in 1992 [24,25,33]. Improvements of human cell engraftment were observed in Rag2 and γ chain double knock-out mice with a mixed background of 129 Ola, Balb/c, and C57BL/6 [34]. BRG (C.Cg-*Rag2^tm1Fwa^Il2rg^tm1Sug^*/JicTac) mice with similar deficiency under BALB/c backgrounds were also reported to support human innate and adaptive immune reconstitution after cord blood-cell-transplantation [29]. Pearson et al. reported the NRG (NOD.Cg-*Rag1^tm1Mom^Il2rg^tm1Wjl^*/SzJ) mice [28], which is one of the most used strains available for constructing humanized mouse models.

For the efficient engraftment of human HSCs, the sub-lethal irradiation of recipient mice is required. This procedure purges or reduces recipients’ HSCs, which would compete with engrafted human HSCs for stem cell factor (SCF). However, the irradiation process deranges bone marrow and the endothelial barriers of recipient mice, and potentially affects the homing of HSCs and immune cells [35,36]. The W/Wv (WBB6F1/J-*Kit^W^/Kit^W-v^*/J) [37] and NBSGW (NOD.Cg-*Kit^W-41J^Tyr^+^Prkdc^scid^ Il2rg^tm1Wjl^*/ThomJ) [30] mice strains are options for the irradiation-free hu-mice construction processes, as the mutation in c-Kit hampers the response of recipients’ HSCs to SCFs.

## 3. Recent Progress in Hu-Mice Models

Though significant improvements have been made to enhance human immune reconstitutions in hu-mice, there are still challenges to using these models for human vaccine development, including the following: (1) human HLA-restricted antigen-specific T cell responses are weak in hu-mice in the context of infection or vaccination; (2) inferior B cell maturation and impairment in IgM to IgG isotype switching in response to infection or vaccination; (3) lymphoid structures, such as B cell follicles and gut-associated lymphoid tissue (GALT), are not fully developed in hu-mice; and (4) the sub-optimal development of certain human immune subsets, especially myeloid cells, in hu-mice. During the last decade, various efforts have been made to overcome these limitations (Table 2).

### 3.1. Improving T Cell Reconstitution and Function through Co-Transplantation of Thymus Tissue or Transgenic Expression of HLA Genes in Recipient Mice

T cell immunity plays a pivotal role in effective antiviral responses, making T cell-based vaccines promising strategies for combating viral infections. The ability of T cells to target conserved viral epitopes [62,63,64] enables them to confer cross-reactivity against diverse strains and variants of viruses and to overcome challenges posed by viral antigenic evolution. The role of T cells in antiviral immunity is well epitomized in a limited number of HIV-1-infected patients called elite controllers or long-term non-progressors, who remain persistently infected without showing marked CD4^+^ T cell depletion in the absence of combined antiretroviral therapy (cART) [65,66,67]. Strong and polyfunctional HIV-1-specific CD4^+^ T cell and CTL responses in these patients are supposed to contribute to the natural control of HIV-1 [68,69]. These observations suggest that T cell-targeting vaccines would offer potential advantages in generating broad and durable protective immunity and hold promise for enhancing vaccine efficacy against highly variable viruses.

The T cell repertoire and function states are largely determined by their development in the thymus, where they are educated by thymus epithelial cells (TECs) and myeloid cells [70]. However, in hu-mice engrafted with human HSCs only, human T cell development is compromised, and the reconstituted T cells are probably educated by both mouse TECs and HSC-derived myeloid cells within the mouse thymus. Thus, it is difficult to induce robust human HLA-restricted antigen-specific T cell responses in hu-mice. One possible remedy is to co-transplant the human thymus along with HSC engraftment. Two groups developed the BLT model, where, in addition to HSCs, the human fetal thymus and fetal liver were co-transplanted into the kidney capsule of NOD-scid mice [33,61]. They showed that human T cells developed in these mice can generate human MHC-I and MHC-II-restricted immunity to Epstein–Barr virus infection and can be activated by human DCs to initiate adaptive responses to superantigens [61]. A subsequent study by Stoddart et al. demonstrated that building BLT mice under an NSG instead of an NOD-scid background resulted in enhanced levels of immune reconstitution and higher susceptibility to intravaginal HIV exposure [71]. Our group generated the NRG-hu Thy/HSC model by transplanting NRG mice with human CD34^+^ HSCs together with fetal thymus tissue, but without fetal liver tissue, from the same donor [72]. As in BLT mice, the human thymic organoid was well developed in NRG-hu Thy/HSC mice and showed long-term sustained thymopoiesis. The NRG-hu Thy/HSC mounted HIV-1-specific T cell responses after infection [73,74].

In addition to the co-transplantation of human thymus tissue, the transgenic expression of human HLA genes is an alternative way to enhance human MHC-restricted T cell responses in hu-mice. Mono-chain HLA class I knock-in hu-mice, which express intact [75,76] or chimeric HLA class I molecules (α1/α2 domain from human HLA, including HLA-A2 and A3 and the α3 domain from mouse H-2D b) [57], enable HLA-I-restricted cytotoxic T lymphocyte (CTL) responses. Mono-chain HLA-A2 knock-in mice mounted an enhanced and HLA-restricted T cell response to Epstein–Barr virus (EBV) [76] and dengue viruses [75], suggesting they could serve as valuable platforms to study vaccine-induced T cell response. Hu-mice with knock-in of HLA class II molecules HLA-DR4 mice were also reported [58]. Serum levels of all four types (IgM, IgG, IgA, and IgE) of natural antibodies in HLA-DR4-expressing hu-mice were comparable to those in humans, making them a precious tool for testing antibody response induced by vaccination [58]. The DRAGA (NSG mice expressing both HLA-A2 and HLA-DR4) mice demonstrated the utilization of the knock-in of both HLA class I and II molecules, as they had been proven to be a model suitable for surrogating the immune response against influenza [59] and SARS-CoV-2 [77]. Similarly, Masse-Ranson et al. reported the BRGSA2DR2 model, which expresses HLA-A2 and HLA-DR2 under Balb/c *Rag2^−/−^Il2rg^−/−^Sirpa^NOD^* (BRGS) mice. The BRGSA2DR2 mice mounted antigen-specific T and B cell responses following the vaccination of the modified vaccinia virus ankara vaccine (MVA-HIVB), which encodes a full-length Gag protein along with partial Pol and Nef antigens [60].

### 3.2. Enhancing B Cell Maturation and LN Formation to Study Vaccine-Induced Antibody Response in Hu-Mice

B cells derived from humanized mice exhibit an immature phenotype characterized by intermediate to high levels of CD24 and CD38, and they also express elevated levels of CD10, another marker indicative of their immature status [78]. The ability of B cells in humanized mice to transition from IgM^+^ to IgG^+^ B cells is limited [7], raising concerns about the reliability of using these models to study humoral immune responses triggered by vaccines. Recent efforts have focused on enhancing the class-switching from IgM to IgG and improving antigen-specific antibody production in these mice. Studies have shown that GM-CSF and IL-4 can boost humoral responses by facilitating the maturation of dendritic cells, B cells, and T cells [50]. Additionally, the introduction of transgenes encoding human stem cell factor, the granulocyte-macrophage colony-stimulating factor, and interleukin-3 has been reported to enhance B cell development in humanized mice [79]. Moreover, Yu et al. showed that hu-mice with human IL-6 knock-in showed enhanced T and B lymphocytes and produced high-affinity and class-switched antibodies with high somatic mutation frequencies after immunization [44], serving as a valuable model to study vaccine-induced B cell responses.

In addition, it is well-known that lymph nodes (LNs), as well as lymphoid structures such as B cell follicles, which are crucial for B cell maturation, somatic mutation, and class-switch, showed development deficiency in hu-mice. This LN development deficiency may be due to defective IL-7 signaling in recipient mice with ablated Il2rg expression, resulting in the absence of lymphoid tissue inducer (LTi) cells, which can promote second lymphoid tissue (SLT) development during the fetal period [80,81,82]. Li et al. developed BRGST mice [55] by inducing thymic stromal lymphopoietin (TSLP) expression in a BRGS mouse model [52]. The BRGST hu-mice successfully developed a full array of LNs with compartmentalized B zones and T zones [55]. Taking advantage of the abundant follicular T cells (T_fh_) in BRGST hu-mice, Li et al. demonstrated that T_FH_ cells are targets of HIV-1 and serve as a reservoir for latent HIV-1 [55]. A recent work by Chupp et al. reported humanized THX-mice by grafting non-γ-irradiated neonate NBSGW (NOD.Cg-*Kit^W-41J^ Tyr^+^ Prkdc^scid^ Il2rg^tm1Wjl^*/ThomJ) or NSGW41 (NOD.Cg-*Kit ^W-41J^ Prkdc^scid^ Il2rg^tm1Wjl^*/WaskJ) mice with human cord blood CD34^+^ cells and conditioning them with a physiologically abundant estrogen, 17β-estradiol, to promote HSC differentiation [56]. They showed that THX mice can generate diverse human B and T cell repertoires and form well-structured lymphoid tissues. The mice mount class-switched, hypermutated, and clonal antibody responses to T cell-dependent and T cell-independent antigens [56]. Moreover, THX mice generated receptor-binding domain (RBD)-neutralizing human antibodies upon being vaccinated intramuscularly with Pfizer BioNTech162b2 coronavirus disease 2019 (COVID-19) mRNA [56], demonstrating their great potential in vaccine development and testing.

### 3.3. Improvement of Graft Efficiency of Certain Human Immune Subsets by Introducing Human Cytokines

With an understanding of the cytokine network involved in human hematopoiesis, researchers started to compensate the human cytokines that are important for the development and homeostasis of certain immune cell types via genetical knock-in (reviewed in refs. [83,84,85]), cytokine-injection, or hydrodynamic injection of cytokine-expressing plasmids [86]. For instance, given the engrafted human HSCs resident in suboptimal niches, where their maintenance, proliferation, and differentiation are disturbed [87], Rongvaux et al. knocked-in thrombopoietin (TPO), which has been demonstrated to be crucial for the maintenance and self-renewal of HSCs [38], and observed increased humanization within the bone marrow of recipient mice. The granulocyte colony-stimulating factor (G-CSF) and the macrophage colony-stimulating factor (M-CSF), based on their role in the development of myeloid cells, were administered to enhance myeloid lineage commitment [39,40].

The combinational supplementation of cytokines was reported as well. While enhanced human hematopoiesis was observed in both studies, Willinger et al. proved that knocking-in both IL-3 and GM-CSF improved human myeloid cells, including human alveolar macrophages [48]; meanwhile, Billerbeck et al. found that the supplementation of stem cell factors (CSFs), GM-CSF, and IL-3, increased CD4^+^FoxP3^+^ regulatory T cell population [49]. Moreover, Rongvaux et al. developed MITRG (M-CSF, IL-3, GM-CSF, TPO) mice, where four genes expressing cytokines important for innate immune subset development were knocked into their respective mouse loci. These cytokines support the development and function of human monocytes, macrophages, and NK cells [51]. In addition, the supplementation of IL-7 [88], IL-2 [42], and IL-15 [41], or the combination of IL-7 and IL-15 [43], enhanced T lymphocyte and NK cell development.

DCs play a crucial role in inducing the T cell response to vaccination. However, human DCs from hu-mice lack the expression of CD209 (DC-SIGN), which is critical for capturing antigens and priming T cells [50]. Chen et al. expressed GM-CSF and IL-4 in hu-mice models via hydrodynamic injection and found that human CD209^+^ DCs were significantly increased, whereby they were able to produce significant levels of neutralizing antigen-specific IgG after H5N1 influenza immunization [50]. One attempt to increase human DC development in hu-mice models is to harness the FMS-like tyrosine kinase 3 (FLT3) signaling. The treatment of the FLT3-ligand (FLT3-L), the ligand for FLT3, was reported to boost DC development in hu-mice [45] and this could be further enhanced in FLT3-deficient mice [46], as the interaction between FLT3 and FLT3-L was reported to have no species specificity [89]. Pham et al. showed that the Flt3-L-mediated expansion of human plasmacytoid dendritic cells inhibits HIV-1 infection in humanized NSG mice [90]. In addition to promoting the development of DCs and T cell priming, the FLT3-L treatment was also reported to enhance the homeostasis of human NK cells and innate lymphoid cells (ILCs) in Balb/c *Rag2^−/−^Il2rg^−/−^Sirpa^NOD^Flk2^−/−^* (BRGSF)-hu HSC mice [47].

Interactions between signal regulatory protein alpha (SIRPα) on macrophages and CD47 on target cells prevent the phagocytosis of target cells by macrophages [91,92,93]. Interestingly, mouse Sirpα polymorphism was found to affect human immune cell accommodation in different mice strains: NOD Sirpα binds to human CD47 at a higher affinity than those of C57BL/6 or Balb/c due to a 20-amino-acid difference in the IgV-like domains [93]. As a result, NOD-scid mice showed superior human hematopoiesis when engrafted with human bone marrow or cord blood cells compared to *Rag2^−/−^*, *Rag2^−/−^Prf1^−/−^*, or *Rag2^−/−^B2m^−/−^* mice under a C57BL/6 background. Inspired by this, Legrand et al. enforced mouse CD47 expression into human progenitor cells and observed increased human NK, T, and B cell reconstitution as well as increased plasma IgM and IgG concentrations [52]. Herndler-Brandstetter et al. knocked both human Sirpα and IL-15 in *Rag2^−/−^Il2rg^−/−^* 129xBalb/c mice, generating SRG-15 mice, in which NK cells infiltrated Burkitt’s lymphoma xenografts and inhibited tumor growth without pre-activation, serving as a valuable tool for studying NK cell-targeted therapies [53]. The reconstitution of human immune system in MITRG mice mentioned above could also be improved by human Sirpα knock-in (termed MISTRG mice) [51]. Moreover, Sungur et al. further improved the MISTRG mice by introducing IL-6 and IL-15 (MISTRG-6-15 mice); these mice demonstrated rapid and functional NK cell responses to HIV-1 infection [54], which was related to vaccine-induced anti-HIV-1 immunity [94].

## 4. Developing Vaccines for HIV-1 Using Humanized Mouse Models

HIV-1 can establish persistent infection in hu-mice, resulting in CD4^+^ T-cell depletion, hyper-inflammatory activation, and immune exhaustion, which mimics the pathology in HIV-1-infected patients. We and others have used the hu-mice models to functionally define the roles of multiple immune cells, including plasmacytoid dendritic cells (pDCs) [95,96,97,98,99], T_fh_ cells [55,100,101], regulatory T cells (Treg) [102,103], and cytokines, including type I interferon [73,74,98] in HIV-1 immuno-pathogenesis. Therefore, hu-mice are proven to be relevant and optimal models for studying HIV-1 persistence, immunopathogenesis, and therapy (Table 3).

An early study by Yoshida et al. showed that the injection of inactivated HIV-1-pulsed autologous DCs into the spleens of SCID-hu PBL mice provides protection against R5 HIV-1 infection [6]. Using the NRG-hu Thy/HSC model, we proved that a T cell-based vaccine which consists of five conserved regions of HIV-1 peptides fused to a CD40 antibody, combined with TLR ligands (TLR-Ls), as an adjuvant, could elicit an anti-HIV-1 CTL response in vivo [107]. Interestingly, we show that R848 and Poly I:C, which can significantly activate human conventional DCs (cDCs), induced a CTL response to the CD40-targeting HIV vaccine [107]. In contrast, CpG-B, which stimulated human pDCs but not cDCs, only enhanced the CD4^+^ helper T cell response [107] or IgG response [8,10], but not the CTL response to the vaccine in hu-mice. Importantly, we tested whether the induction of an anti-HIV CTL response could have therapeutic effects on hu-mice with persistent HIV-1 infection. We showed that when administrated in therapeutic settings in HIV-1-infected hu-mice, under effective highly active antiretroviral therapy (HAART), the aCD40.HIV5pep with poly(I:C) vaccination reduced the level of cell-associated HIV-1 DNA in lymphoid tissues. Most importantly, the vaccination significantly delayed the rebound of HIV-1 after HAART cessation [9]. A recent piece of research also showed vaccination in BLT mice with poly I:C and STING agonist-primed DC loaded with a pool of HIV GAG peptides increased polyfunctional HIV-1-specific CD8^+^ T cells in lymphoid tissue and reduced HIV-1-induced CD4^+^ T cell depletion in vivo [13]. Norton et al. developed a lentiviral vector-based DC vaccine and proved that the injection of BLT mice with DCs transduced with the vector expressing HIV-1 SL9 epitope and that CD40L triggers the antigen-specific T cell response and suppresses HIV replication by two logs for 6 weeks in vivo [11]. Claiborne et al. explored the utility of the BLT mice for HIV-1 vaccine development by immunizing mice against the conserved Gag protein of HIV-1. They utilized a rapid prime-boost protocol of poly(lactic-co-glycolic) acid microparticles and a replication-defective herpes simplex virus recombinant vector and found that the immunization procedure redirects Gag-specific T cell responses and reduces viral load after HIV-1 infection. In addition, Xu et al. developed a short carbon nanotube-based delivery platform which can co-deliver HIV-1 glycoproteins and HIV-1 mRNA to the immune system efficiently. They proved that the candidate vaccine induced humoral and cellular responses in humanized NSG-B2m mice (NOD.Cg-B2^mtm1Unc^Prkdc^scid^ Il2rg^tm1Wjl/SzJ^) that express HLA-A2, HLA-DR4, IL-3, Il-4, IL-6, IL-7, IL-15, and GM-CSF [12]. Moreover, they showed that one-third of the infected hu-mice vaccinated with the NanoVac–mRNA was cleared of HIV-1 infection by 8 weeks post-infection [12].

It was reported that the induction of antigen-specific IgG responses is weak in hu-mice [108] due to the fact that B cells developed in hu-mice are immature [7,44,50,78,79,108]. Biswas et al. show that the intramuscular immunization of BLT mice with HIV-1 gp140 or West Nile Virus envelope proteins induced antigen-specific human antibodies, predominantly of the IgM isotype [7]. Studies have suggested that a lack of human cytokines and the disorganized secondary lymphoid structures might lead to defects in B cells [109,110,111]. We showed that stimulation of Toll-like receptors (TLRs) results in significant cytokine induction in hu-mice [107]. Like in humans, the activation of pDC by the TLR-9 agonist CpG-B in vivo in hu-mice induces IFN-α as well as IL-6 production [107]. It was reported that pDC-derived IFN-a and IL-6 were critical for IgG induction from human PBMCs in response to influenza virus [112]. Inspired by this, we developed a CD40-targeting HIV-1 vaccine combined with CpG-B as an adjuvant. We proved that the vaccination strategy can induce B cell maturation and promote IgG induction in a pDC-dependent manner in hu-mice [8]. Meanwhile, the CD40-targeting HIV-1 vaccine, alone, and CpG-B plus the non-CD40 targeting recombinant protein did not induce a significant IgG response [8]. Moreover, we showed that CD40-targeted HIV-1 Env vaccination with CpG-B as an adjuvant induced rigorous expansion, somatic hypermutation, and the IgG switch of HIV-1 gp140-specific human B cells in hu-mice, whose magnitude was correlated with the proportion of circulating memory ICOS^+^ follicular helper T cells [10]. In addition, Buffa et al. also reported that sublingually administrate of HIV-1 CN54gp140 with CpG-B-adjuvant induced IgG and IgA in the vaginal mucosa [113], implying protection from HIV-1 infection through sexual transmission.

Since inducing high-affinity IgG by vaccination in hu-mice still faces difficulties, researchers have also developed humanized immunoglobulin mice models for HIV vaccine testing (reviewed in ref. [114]). Recently, Xie et al. reported that a sequential administration of a priming HIV-1 immunogen (N332-GT5, V3-glycan-targeted) and two boosted immunogens (B11 and B16) intravenously induced long-lasting antibody responses with high somatic hypermutation and affinity in immunoglobulin knock-in hu-mice [16]. Later research by this group reported that the VRC01-class switch, a characteristic of broadly neutralizing antibodies (bnAbs), could be induced by mRNA-LNP vaccines encoding eOD-GT8 60mer in humanized immunoglobulin mice [15]. Besides, through the intramuscular delivery of the same epitope into humanized immunoglobulin mice, Chen et al. identified two promising bnAbs that showed great breadth (>50% breadth on a 208-strain panel) [104]. In addition to generating VRC01-class antibodies, targeting the CD4-binding site, attempts to design and test germline-targeting vaccines that could induce bnAbs, targeting the V2-apex [106] and membrane-proximal external region (MPER) [105] of HIV-1 Env spikes, were also reported. The mRNA-LNP HIV-1 vaccines were evaluated in a Phase I clinical trial (NCT05001373), demonstrating the value of humanized immunoglobulin mice models in bridging the information gap between preclinical and clinical vaccine assessments.

Collectively, these findings indicated that hu-mice with human immune systems or humanized immunoglobulin mice are powerful tools in investigating anti-HIV-1 immunity, and the development and testing of anti-HIV-1 vaccines.

## 5. Developing and Testing Vaccines against Other Pathogens in Humanized Mouse Models

Humanized mouse models are also instrumental in the development and testing of vaccines against diverse viruses. Influenza, like HIV-1, are RNA viruses with unstable genomes that can mutate and evolve rapidly to escape antibody neutralization [115,116,117]. As a result, annual vaccination against different influenza strains is required to provide proper immunity to the predicted pandemic strain. With the cross-reactive anti-influenza T cells reported [118,119], researchers turned to developing T cell-based vaccines against influenza using hu-mice models (reviewed in detail in ref. [120]). Graham et al. developed DC-targeting influenza matrix protein-1 (FluM1) vaccines and found that they induced stronger antiviral responses in hu-mice than in vitro, demonstrating the utility of hu-mice in T cell-based vaccine development and testing [121]. In addition to DC-targeted antigen delivery, multiple T cell-based vaccination strategies, including DNA vaccines [122] and DNA-protein chimeric vaccines [120], were tested in hu-mice models as well. Other than viruses, vaccines against other types of pathogens were also tested in hu-mice models. For instance, Malaria caused by Plasmodium parasites is another significant global health burden, and there is no FDA-approved malaria vaccine yet. Using the DRAGA mice, Majji et al. proved that live P. falciparum sporozoites under chloroquine chemoprophylaxis would induce P. falciparum sporozoite-specific T and B cell responses [17], demonstrating the utilization of hu-mice as a pre-clinical model to evaluate malaria vaccine candidates.

## 6. Conclusions

In summary, humanized mouse models have significantly advanced the field of vaccine research by offering a more accurate representation of human immune responses compared to traditional animal models. These models have been instrumental in studying the complexities of human T and B cell responses to pathogens like HIV-1. Despite these advances, challenges remain, including the incomplete reconstitution of human immune cells, suboptimal lymphoid organ development, and the need for better modeling of human-specific diseases. As these models evolve, they hold great promise for bridging the gap between preclinical studies and clinical applications, ultimately contributing to the development of effective vaccines for challenging infectious diseases.

## Figures and Tables

**Table 1:** Immunodeficient mice used for hu-mice construction.

Common Name	Full Name	Characteristics	References
NOD scid	NOD.Cg-*Prkdc^scid^*/J	Lacks functional T and B cells due to Prkdc mutation, compromised NK cell and macrophage function	[20]
Rag1 KO	B6.129S7-*Rag1^tm1Mom^*/J	Lacks functional T and B cells due to Rag1 mutation	[24]
Rag2 KO	B6.Cg-*Rag2^tm1.1Cgn^*/J	Lacks functional T and B cells due to Rag2 mutation	[25]
NOG	NOD.Cg-*Prkdc^scid^ Il2rg^tm1Sug^*/Jic	Lacks T cells, B cells, and NK cells; compromised NK cell and macrophage function	[26]
NSG	NOD.Cg-*Prkdc^scid^ Il2rg^tm1Wjl^*/SzJ	Lacks T cells, B cells, and NK cells; compromised NK cell and macrophage function	[27]
NRG	NOD.Cg-*Rag1^tm1Mom^ Il2rg^tm1Wjl^*/SzJ	Lacks mature T cells, B cells, and NK cells; compromised NK cell and macrophage function	[28]
BRG	BALB/c-*Rag2^−/−^Il2rg^−/−^*	Lacks mature T cells, B cells, and NK cells	[29]
NBSGW	NOD.Cg-*Kit^W-41J^Tyr^+^Prkdc^scid^Il2rg^tm1Wjl^/*ThomJ	Lacks mature T cells, B cells, and NK cells; irradiation-free before human HSC engraftment due to the Kit mutation	[30]

**Table 2:** Recent progress in improving hu-mice models.

Human Cytokines/Hormones Supplementation		
Cytokine	Key Findings	References
TPO	Increased myelomonocytic/lymphoid lineages ratio and HSC maintenance	[38]
M-CSF	Increased development of myeloid cells in hematopoietic organs and enhanced monocytes/macrophage phagocytosis function	[39]
G-CSF	Improved the development of mature monocytes and tissue-resident macrophages; mounted enhanced protection against influenza virus and Mycobacterium infection	[40]
IL-15	Improved NK cell development; robust NK cell response to HIV-1	[41]
IL-2	Human NK cell receptors and effector molecule expression comparable to levels in humans; rejection or suppression of leukemia cell lines inoculated	[42]
IL-7 and IL-15	Increased frequencies of human NK cells in multiple organs	[43]
IL-6	Enhanced thymopoiesis and periphery T cell engraftment; increased class-switched memory B cells IgG; produced high-somatic-mutation-rate antibodies	[44]
Flt3-L	Boosted conventional DC and plasmacytoid DC development, which responds to TLR agonists and DC-targeting vaccination; increased numbers of human NK and T cells; improved human NK and ILC homeostasis	[45,46,47]
GM-CSF+IL-3	Increased myeloid cell frequencies; alveolar macrophages mounted a human-like response to influenza	[48]
SCF+GM-CSF+IL-3	Elevated myeloid cell frequencies including myeloid dendritic cells; increased functional CD4^+^FoxP3^+^ regulatory T cells	[49]
GM-CSF+IL-4	Development of CD209^+^ DCs; produced significant levels of neutralizing IgG following H5N1 influenza immunization	[50]
M-CSF, IL-3, GM-CSF, TPO, with or without Sirpα (MITRG/MISTRG)	Improved monocytes, macrophages, and NK cells development; macrophages infiltrated to human tumor xenograft	[51]
Sirpa (BRGS)	Improvement of progenitor cell engraftment and human T, B, and NK cell homeostasis; elevated plasma IgM and IgG concentrations	[52]
IL-15 and Sirpα	Development and functional maturation of circulating/tissue-resident human NK and CD8^+^ T cells; increased ILC development	[53]
IL-6 and IL-15	Quick NK cell response to HIV-1 in non-lymphoid organs	[54]
TSLP (BRGST)	Full array of lymph nodes (LNs); larger thymus; more mature B cells and T follicular helper cells	[55]
17beta-estradiol (THX)	Fully reconstituted human lymphoid and myeloid immune system, well-formed LNs, and intestinal lymphoid tissue; mount neutralizing antibody responses to vaccination	[56]
Expressing human leukocyte antigen (HLA)		
HLA genes	Key findings	References
HLA-A	HLA-restricted, epitope-specific CTLs were induced upon vaccination of various viruses	[57]
HLA-A2402 and HLA-A0301	CTL response against both HLA-A24 and HLA-A3 epitopes when vaccinated with a mixture of both peptides	[57]
HLA-DR4	Reconstituted human-like Ig serum levels; elicited high titers of specific human IgG antibodies following tetanus toxoid vaccination	[58]
HLA-A2 and HLA-DR4 (DRAGA)	Serum natural Ig levels were comparable to humans; higher IgG titer upon tetanus toxoid vaccination; generated neutralizing antibodies after vaccinated with KLH-conjugated influenza hemagglutinin epitope; administration of neutralizing antibodies reduced the lethality rate and lung damage in influenza-infected mice	[59]
HLA-A2 and HLA-DR2 (BRGSA2DR2)	Enhanced T cell development in the thymus; accelerated T cell emergence into circulation; enhanced antigen-specific T and B cell response following MVA-HIVB vaccination	[60]
Co-transplantation of human thymus		
Co-transplantation of fetal thymus and liver	Systemic and comprehensive reconstitution of human lymphohematopoietic cells; generated HLA class I- and HLA class II-restricted T cell response to EBV infection; systemic human Vβ2^+^ T cell expansion after superantigen toxic shock syndrome toxin 1 administration; produce high levels of human IgM and IgG antibodies; rejection to skin xenograft	[33,61]

**Table 3:** Developed and tested vaccines in hu-mice and humanized immunoglobulin mice models.

Vaccine Design	Adjuvant	Administration	Model	Key Findings	References
Mature DCs pulsed with inactivated HIV-1	-	Intrasplenic	SCID-hu PBL	Induced antigen-specific T cell immune response; sera from immunized mice inhibited HIV-1 infection of PBMCs and macrophages in vitro	[6]
Recombinant HIV-1 envelope gp140 antigen	IC31	Intramuscular	BLT	IgM predominated antigen-specific human antibodies; CD19^+^ CD5^+^ instead of CD19^+^ CD5^−^ B cell displayed memory phenotype	[7]
Anti-CD40 antibody with five conserved HIV-1 epitopes fused to heavy chain C-terminus	CpG-B	Intramuscular (half dose) and intraperitoneal (half dose)	NRG-hu HSC	Induced mature IgG^+^ B cells; induced significant levels of antigen-specific IgG	[8]
Anti-CD40 antibody with five conserved HIV-1 epitopes fused to heavy chain C-terminus	Poly(I:C)	Intramuscular (half-dose) and intraperitoneal (half-dose)	NRG-hu HSC	Induced antigen-specific T cell response; reduced HIV-1 reservoir; delayed HIV-1 rebound after HAART cessation	[9]
HSC-derived DCs incubated with Gag peptide	2′3′-c′diAM(PS)2 and Poly I:C	Intravenous	BLT	Reduced CD4^+^ T-cell depletion following HIV-1 infection and reduced HIV-1^+^ cell spreading to LN; preserved CD8^+^ T cells polyfunctionality	[13]
Anti-human CD40 antibody fused to the gp140ZM96 Clade C protein to the heavy chain C-terminus	CpG-B	Intravenous	NRG-hu HSC	Induced IgG^+^ B cells with broad Ig VH/VL repertoires and high somatic mutation rate; Induced splenic GC-like structures containing human B cells and PD-1^+^ BCL6^+^hu-Tfh-like cells	[10]
HSC-derived DCs expressing CD40L and HIV-1 SL9 epitope	-	Intravenous	HLA-transgenic BLT	Induced antigen-specific T cell proliferation and memory differentiation; reduced viral load by two logs for 6 weeks	[11]
Prime: Gag-specific poly(lactic-co-glycolic) acid Boost: Gag-expressing, replication-defective herpes simplex virus 1 (HSV-1) vector	-	Intravaginal or intraperitoneal	HLA-transgenic BLT	Induced Gag-specific T cell responses; reduced viral load immunized mice after infection	[14]
Short carbon nanotube-based co-delivery of HIV-1 epitope V1V2 (ZM53)-2F5K-encoding mRNA and HIV-1 Glycoprotein	-	Intramuscular or intranasal	NSG-B2m-hu HSC mice expressing HLA-A2, HLA-DR4, IL-3, Il-4, IL-6, IL-7, IL-15, and GM-CSF	Induced antigen-specific cellular and humoral response; 33% immunized mice were virus-free by 8 weeks post-infection	[12]
Trimer immunogen N332-GT5, B11 and B16	-	Intramuscular	Human BCR-expressing C57BL/6J	Generated durable GCs, BG18 B cells with somatic hypermutation, and affinity maturation	[16]
eOD-GT8 60mer mRNA-LNP	-	Intramuscular	Human BCR-expressing C57BL/6J	Evolved B cell precursors toward VRC01-like broadly neutralizing antibodies	[15]
eOD-GT8 60mer nanoparticle	-	Intramuscular	Human BCR-expressing C57BL/6J	Generated VRC01-class antibody precursors; identified VRC01-class bnAbs, including with >50% breadth on a 208-strain panel	[104]
MPER-HuGL18 nanoparticle	-	Intraperitoneal	Human BCR-expressing C57BL/6J	Long-term GC residency and maturation of MPER-HuGL18 precursors	[105]
ApexGT5 mRNA-LNP	-	Intraperitoneal or intramuscular	Human BCR-expressing C57BL/6J	Increased activation and recruitment of PCT64 precursors to GCs and lowered	[106]
